# Metformin alleviates choline diet-induced TMAO elevation in C57BL/6J mice by influencing gut-microbiota composition and functionality

**DOI:** 10.1038/s41387-021-00169-w

**Published:** 2021-07-31

**Authors:** Chunyan Su, Xingxing Li, Yuxin Yang, Yu Du, Xiumin Zhang, Li Wang, Bin Hong

**Affiliations:** 1 NHC Key Laboratory of Biotechnology of Antibiotics, Chinese Academy of Medical Sciences & Peking Union Medical College, No.1 Tiantan Xili, Beijing, 100050 China; 2 CAMS Key Laboratory of Synthetic Biology for Drug Innovation, Institute of Medicinal Biotechnology, Chinese Academy of Medical Sciences & Peking Union Medical College, No.1 Tiantan Xili, Beijing, 100050 China

**Keywords:** Microbiology, Cardiovascular diseases

## Abstract

Trimethylamine-N-oxide (TMAO), a gut-microbiota-dependent metabolite generated from its dietary precursors such as choline, has been identified as an independent risk factor for atherosclerosis. Metformin is the most widely used drug for the treatment of type 2 diabetes (T2D), which has therapeutic effects on hyperglycemia accelerated atherosclerosis. A growing body of evidence suggest that metformin plays a therapeutic role by regulating the structure and metabolic function of gut microbiota. However, whether metformin has an impact on gut-microbiota-mediated TMAO production from choline remains obscure. In this study, the oral administration of metformin significantly reduced choline diet-increased serum TMAO in choline diet-fed C57BL/6J mice. The diversity analysis based on 16S rRNA gene sequencing of C57BL/6J mice fecal samples indicated that metformin markedly changed the gut-microbiota composition. Metformin was positively correlated with the enrichment of different intestinal bacteria such as *Bifidobacterium* and *Akkermansia* and a lower *cutC* (a choline utilization gene) abundance. Furthermore, the ex vivo and in vitro inhibitory effects of metformin on choline metabolism of TMA-producing bacteria were confirmed under anaerobic condition. The results suggested that metformin suppresses serum TMAO level by remodeling gut microbiota involved in TMA generation from choline.

## Introduction

Reducing atherosclerosis burden in type 2 diabetes (T2D) patients is a major clinical imperative to reduce mortality and morbidity and to improve life quality. Metformin is one of the first-line T2D therapeutic agents all over the world, which has been given to millions of patients for more than 60 years considering its low cost and relatively good tolerance [1]. In recent years, growing evidence has shown that gut-microbiota dysbiosis can play a major role in the development of T2D, and that some clinical benefits of metformin may be mediated by gut microbiome, dependent and independent of its antidiabetic effect. Forslund *et al*. reported a unified signature of gut microbiome shifts in T2D, and that the therapeutic effects of metformin might be partly attributed to gut-microbiota-mediated short-chain fatty acid production [2]. Sun et al. described that metformin could alter the gut-microbiota composition and bile acid metabolism, subsequently influencing host metabolism through the intestinal FXR signaling pathway [3]. Intriguingly, metformin was identified to regulate host metabolism and longevity through gut microbial agmatine production under the control of a bacterial metabolic signaling axis integrating drug-nutrient-microbiome interactions [4]. All these researches imply an importance to disentangle various clinical beneficial effects of metformin from the perspective of gut microbiome.

Strong evidence has been found that the gut microbiota, particularly microbiome-derived metabolites, play a pivotal role in the development of cardiovascular diseases [5]. Numerous human and animal studies support that the serum trimethylamine-N-oxide (TMAO) from gut-microbiota-derived trimethylamine (TMA) is positively related to atherosclerosis risk, such as through suppression of reverse cholesterol transport [6]. Elevated circulating TMAO may also accelerate thrombus formation by enhancing platelet responsiveness, and deteriorate vascular endothelial cell senescence and vascular inflammation [7, 8]. The dietary provision of TMA-containing nutrients such as choline, carnitine and phosphatidylcholine can promote atherosclerosis, and circulating TMAO level increases with age in both humans and mice [7, 9]. Furthermore, higher serum TMAO level was revealed closely associated with cardiovascular risk in T2D patients [10]. Thus, the TMA or TMAO generation was proposed to be a potential therapeutical target by “drugging” gut microbiota that produces TMA. Several designed specific CutC/D inhibitors, such as 3,3-dimethyl-1-butanol (DMB), betaine aldehyde and halomethylcholines, could suppress TMA production in different human gut microbiota isolates harboring *cutC/D* genes and human fecal suspensions. Plasma TMAO level and atherosclerosis plaque in mice were also inhibited by these compounds [11–13]. Besides, a natural product resveratrol was reported to inhibit TMA and TMAO production from choline via modulating gut microbiota in mice, which retards the atherosclerosis process [14]. It was found that metformin attenuated atherosclerosis and vascular senescence in mice and showed protective effects against vascular disease in T2D patients [15, 16]. However, as a newly identified independent atherosclerotic risk factor, whether TMAO level may be influenced by metformin remains obscure.

According to these findings, we proposed a hypothesis that metformin may play a role in suppressing the progress of atherosclerosis by modulating the TMAO level through the gut microbiota. Here, we examined the effect of metformin on circulating TMA and TMAO levels and the possible contribution of the gut microbiota in high-choline diet-fed C57BL/6J mice. The gut-microbiota structure and abundance of TMA-producing gene *cutC* altered by metformin were investigated. Furthermore, the effect of metformin on choline transformation to TMA by gut microorganisms was observed ex vivo and in vitro. All the results presented in this study provided insight on the impact of metformin on TMA-producing ability of gut microbiota and ultimately the serum TMAO level.

## Subjects and methods

See details in Supplementary Methods.

### Animal and treatments

All animal experiments were performed following the recommendations in the Guide for the Care and Use of Laboratory Animals and were approved by the Institutional Authority for Laboratory Animal Care of Institute of Medicinal Biotechnology. For all experiment, 6- to 8-week-old female C57BL/6J mice (C57BL/6JNifdc, Beijing Vital River Laboratory Animal Technology Co., Ltd) were fed a chow diet or high-choline diet.

### HPLC-MS/MS detection of serum TMA/TMAO level

Samples were analyzed with LC-MS/MS using an Agilent 6400 Series Triple Quadrupole mass spectrometer (Agilent Technologies, Wilmington, DE, USA) equipped with an electrospray ionization source.

### Quantitation of d9-TMA production

The d9-TMA-producing activity of mice fecal sample was quantified ex vivo in anaerobic condition (10% CO_2_–10% H_2_–80% N_2_) using AW400SG anaerobic workstation (Electrotek, West Yorkshire, UK).

### Metagenomic DNA extraction and microbial diversity analysis

FastDNA^®^ SPIN kit for Feces and the FastPrep^®^ Instrument (MP Biomedicals, Santa Ana, CA, USA) were used to extract and purify the metagenomic DNA of mice fecal samples. Extracted DNA was used as template to amplify the V3–V4 region of the bacterial 16S rRNA gene. The amplicon libraries were used for diversity and structural comparisons of the bacterial species by Illumina MiSeq platform (Majorbio Bio-Pharm Technology, Shanghai, China). Bioinformatics analysis was conducted on the Majorbio I-Sanger Cloud Platform (www.i-sanger.com).

### Detection of *cutC* abundance via quantitative real-time PCR (qPCR)

Amplification was performed using TransStart Tip Green qPCR SuperMix (TransGen Biotech, China) according to the manufacturer’s instructions.

### Statistical analyses

After testing the normal distribution and homogeneity of variance, unpaired two-tailed student’s *t*-test was applied for comparing two groups and one-way ANOVA for multiple groups, followed by Tukey’s correction as applicable (GraphPad Software, San Diego, CA, USA). Data were presented as mean ± SEM. *P* value of <0.05 was significant. Statistical differences for the abundance of genus which did not pass normality testing were conducted with Kruskal–Wallis *H* test followed by pairwise comparisons and the data presented as median with interquartile ranges.

## Results and discussions

### Metformin inhibited choline diet increased TMAO level in C57BL/6J mice

**Fig. 1:**
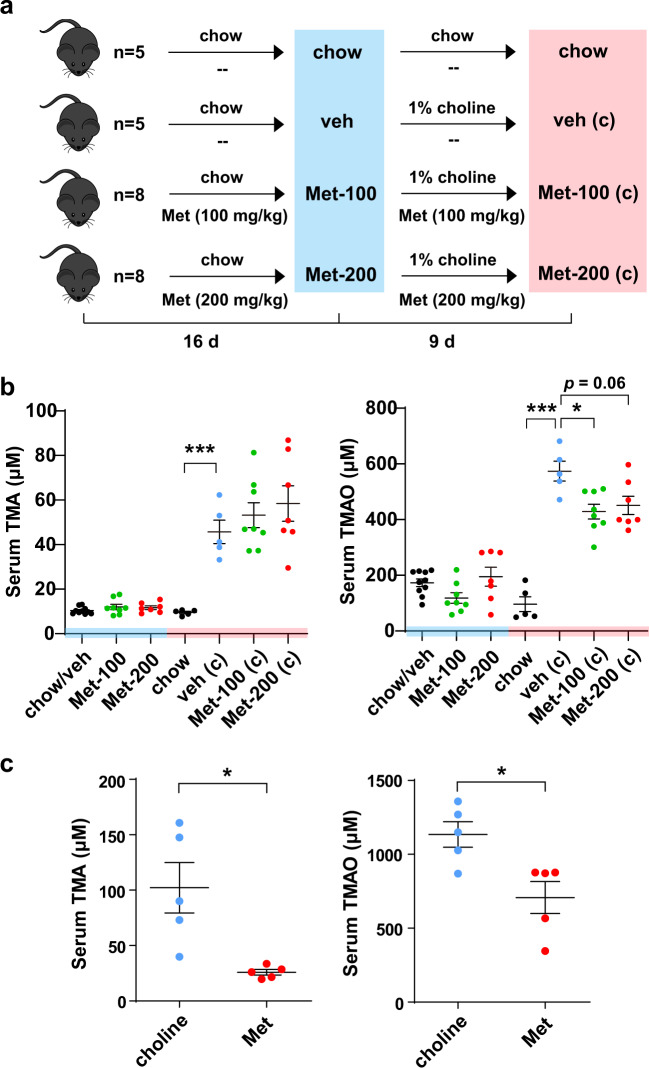
The effects of metformin on serum TMA and TMAO levels in C57BL/6J mice under chow or choline diet. **a**, **b** Female C57BL/6J mice were fed chow with or without 100 mg/kg metformin (Met-100) or 200 mg/kg metformin (Met-200) for 16 days, then fed 1% choline diet with or without metformin (veh (c), Met-100 (c), Met-200 (c)) for another 9 days. Mice administration as illustrated in schematic diagram (**a**). TMA and TMAO levels were detected at indicated time points by HPLC-MS/MS (**b**). veh/veh (c), *n* = 5; chow, *n* = 5; Met-100/Met-100 (c), *n* = 8; Met-200/Met-200 (c), *n* = 8. **c** 2% choline fed C57BL/6J mice were applied to evaluate the effects of metformin on TMA and TMAO levels. C57BL/6J mice were initially fed on chow diet with or without 200 mg/kg metformin for 6 weeks and sequentially converted to 2% choline diet for another 3 weeks. Serum TMA and TMAO levels were determined. *n* = 5 for each group. Values are presented as means ± SEM (*p < 0.05, ***p < 0.001).

We initially tested the effect of metformin in an in vivo study. Chow diet-fed C57BL/6J mice were treated with vehicle, 100 or 200 mg/kg metformin daily by gavage. After 16 d’s administration, we switched the chow diet into 1% choline diet for another 9 days. Serum TMA and TMAO levels were tested before and after the administration of choline (Fig. 1a). It was shown that before the administration of choline serum TMA and TMAO level was not affected by metformin (Fig. 1b). After 9 days’ administration of choline, both TMA and TMAO production was dramatically increased, and the choline-induced TMAO level was significantly reduced by different concentrations of metformin, while TMA level was not altered significantly possibly due to relatively short treatment time (Fig. 1b).

To further confirm the influence of metformin on gut-microbiota-derived TMA and TMAO, an administrative protocol with longer treatment of metformin was performed. Two groups of C57BL/6J mice were daily treated with or without 200 mg/kg metformin for 9 weeks. In the first 6 weeks the mice were fed chow diet, and then fed 2% choline diet in the following 3 weeks. At the end, serum TMA and TMAO were both significantly decreased by metformin compared with vehicle (Fig. 1c). These results indicated that metformin reduced TMAO generation in choline fed C57BL/6J mice.

### Metformin reversed the choline-induced gut-microbiota dysbiosis

**Fig. 2:**
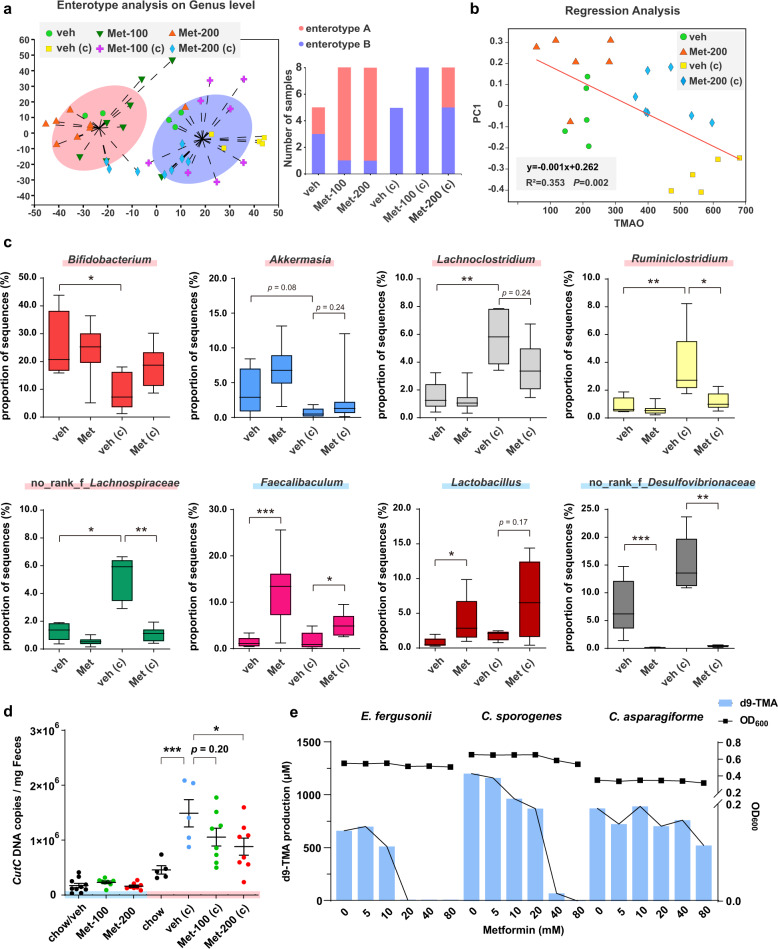
Metformin treatment altered composition of gut microbiota and inhibited TMA production from choline. **a** Effects of metformin on enterotype of C57BL/6J mice fed with chow or choline diet. Samples were calculated using Jensen-Shannon distance and separated into two clusters by the PAM method. The left panel showed the clustering of two top principal components. **b** Linear regression analysis of the correlation between serum TMAO level and microbiota beta diversity based on OTU levels with bray curtis algorithm. **c** The abundance of significant different genera among four groups. Metformin, 200 mg/kg. Boxes show the median with interquartile ranges, whiskers show the minimum and maximum values. *p*-values are from Kruskal–Wallis H test (*p < 0.05, **p < 0.01, ***p < 0.001). **d** The abundances of *cutC* gene in fecal samples were measured by qPCR. veh/veh (c), *n* = 5; chow, *n* = 5; Met-100/Met-100 (c), *n* = 8; Met-200/Met-200 (c), *n* = 8. The error bars represented the mean ± SEM (*p < 0.05, ***p < 0.001). **e** Effects of metformin on TMA-producing bacteria under anaerobic condition. The production of d9-TMA from d9-choline (bar) and OD_600_ (line) were measured after metformin treatment for 24 h.

Dietary choline is metabolized to TMA by the gut microbiota, and then TMA is transformed to TMAO in the liver, dependent on flavin monooxygenase (FMO) enzyme family, among which FMO3 is most relevant [17]. Thus, we firstly examined the effects of metformin on FMO3 expression in the liver of choline fed mice and found that metformin treatment hardly changed FMO3 protein level (Supplementary Fig. 1). Then we sought to explore the correlation between the gut microbiota and the metformin-derived alleviation of the diet-induced increase in serum TMAO. We collected 52 fecal samples of mice in Fig. 1a before and after choline administration, and the fecal microbiota was profiled with 16 S rRNA gene high-throughput sequencing. Clustering of the fecal microbiomes using a partitioning around medoids (PAM) algorithm showed two distinct clusters (i.e., enterotypes) based on their genus level compositions, named as “enterotype A” and “enterotype B”, enriching *Bifidobacterium* or f_*Muribaculaceae* respectively (Fig. 2a) [18]. It can be seen that in the three groups under chow diet (veh, Met-100 and Met-200) both enterotypes existed, and the proportion of enterotype-A samples increased when administrated with metformin. The choline diet increased the proportion of enterotype-B samples when compared with normal dietary conditions. High dosage of metformin reversed the choline-induced enterotype switch, suggesting metformin may have a protective effect on the gut microbiota (Fig. 2a).

Then the microbiota compositions between vehicle and metformin treatment groups (Met-200) under normal or choline diet were analyzed and compared through principal coordinate analysis (PCoA) at OTU level, and a significant difference in microbiota composition was found within the four groups (Supplementary Fig. 2a). A linear regression analysis indicated that the fecal microbial community exhibited a significant correlation with serum TMAO level (Fig. 2b). As shown in Supplementary Fig. 2b, the microbiota contained 27 predominant genera with relative abundance of more than 1% in all samples, and these genera were subjected to investigate the correlation between bacterial abundance alteration and the mice phenotypes by Spearman’s correlation analysis (Supplementary Fig. 2c). Notably, the relative abundance of *Bifidobacterium* and *Akkermansia* were decreased in the high-choline diet group and restored by metformin administration (Fig. 2c), which were negatively correlated with TMAO level. On the contrary, the relative abundance of *Lachnoclostridium*, *Ruminiclostridium* and no_rank_*Lachnospiraceae* were increased significantly after choline diet and reversed by metformin, which exhibited positive correlation with TMAO level (Fig. 2c, Supplementary Fig. 2c). Consistently, growing evidence suggest that the abundance of genera *Bifidobacterium* and *Akkermansia* decrease in T2D patients, and metformin treatment may reverse their relative abundance [2, 19]. Recent studies have also shown that oral administration of *Akkermansia muciniphila*, a well-documented mucin-utilizing species in this genus, significantly improved insulin sensitivity and reduced cholesterol levels in obese human volunteers [20].

Additionally, among these predominant genera metformin-treated mice had a significant higher proportion of *Faecalibaculum* and *Lactobacillus* and significant lower abundance of no_rank_f_*Desulfovibrionaceae* compared to their respective vehicle groups. However, these genera showed weak correlation with TMAO level, suggesting that they may be related to other physiological effects of metformin (Fig. 2c, Supplementary Fig. 2c). In fact, known as traditional probiotics, *Lactobacillus* has previously been shown to exert antidiabetic and cholesterol-lowering efficacy in rodents [21, 22]. Furthermore, metformin could restore glucose sensing while increasing the abundance of *Lactobacillus* in the upper small intestine, which is reduced in HFD-induced rodents [23].

### Metformin suppressed *cutC* gene abundance

The microbial choline TMA-lyase CutC, a glycyl radical enzyme, was found to cleave C–N bond of choline to produce TMA in various bacteria, and the correlation between CutC and TMA production has also been well documented [24]. To further investigate the mechanism underlying metformin induced decreases of serum TMA and TMAO levels, the abundance of *cutC* gene in fecal samples was determined by qPCR. The results revealed that the *cutC* gene abundance showed significant positive correlations with serum TMA and TMAO levels (Supplementary Fig. 2d). Furthermore, the *cutC* gene abundance increased significantly after choline diet, which was markedly decreased by metformin treatment (Fig. 2d), suggesting a pivotal role of gut microbiota in production of TMA regulated by metformin.

### Metformin inhibited TMA formation anaerobically ex vivo and in vitro

To confirm the inhibitory effect of metformin on choline-to-TMA transformation, the fecal microbiota from chow diet C57BL/6J mice were investigated in an ex vivo assay cultured with deuterium-labeled choline (d9-choline). After 14 h of anaerobic incubation, d9-TMA production decreased in a dose-dependent manner with increasing metformin concentrations (Supplementary Fig. 3). The IC_50_ (50% inhibitory concentration) of metformin for two pooled fecal samples from different cages of mice were 1286 µM and 347.7 µM, respectively.

Subsequently, three reported TMA-producing human commensal strains including *Clostridium asparagiforme*, *Clostridium sporogenes* and *Escherichia fergusonii* [25] were cultured in vitro to assess the inhibitory effect of metformin on TMA transformation from choline. The results showed that metformin had different inhibitory effects on TMA production of these strains in a nonlethal manner (Fig. 2e). Specifically, *E. fergusonii* seemed more sensitive to metformin administration, which completely inhibited TMA production at a concentration of 20 mM, while the growth was hardly affected at the same concentration (Fig. 2e). These results indicated that metformin has the potential to inhibit TMA production of some TMA-producing strains, especially in the context of the gut microbiota. It is noteworthy that the oral bioavailability of metformin is approximately 50%, and the peak concentration in the intestinal tract could be up to 300-fold greater than that in plasma [26], suggesting the clinical relevance of the ex vivo and in vitro results although at the mM range of concentration of metformin.

Taken together, these results showed that metformin may decrease TMA production by remodeling gut microbiota and suppressing the abundance of choline metabolizing gene *cutC*, thus reducing serum TMAO level, an independent atherogenic factor. This finding suggests a novel mechanism of metformin action through influencing the TMA production of gut microbiota, which may explain at least part of its therapeutic effects in reducing the cardiovascular risk of T2D patients.

## Supplementary information

Supplementary material
