# Supplementary material for: Metformin alleviates choline diet-induced TMAO elevation in C57BL/6J mice by influencing gut-microbiota composition and functionality
Source: Nutr Diabetes. 2021 Jul 31;11:27. doi: 10.1038/s41387-021-00169-w (PMC8363624; doi:10.1038/s41387-021-00169-w)
Supplement: Supplementary file 1 — Supplementary material [file 41387_2021_169_MOESM1_ESM.docx]

**Supplementary material**

**Metformin alleviates choline diet induced TMAO elevation in C57BL/6J mice by influencing gut microbiota composition and** **functionality**

Chunyan Su^a1^, Xingxing Li^a,b1^, Yuxin Yang^a^, Yu Du^a^, Xiumin Zhang^a^, Li Wang^a^*, Bin Hong^a,b^*

^a^NHC Key Laboratory of Biotechnology of Antibiotics, ^b^CAMS Key Laboratory of Synthetic Biology for Drug Innovation, Institute of Medicinal Biotechnology, Chinese Academy of Medical Sciences & Peking Union Medical College, No.1 Tiantan Xili, Beijing 100050, China.

^1^These authors contributed equally to this article.

*To whom correspondence should be addressed.

Email: wangli_imb@163.com, wangli@imb.pumc.edu.cn (Li Wang);

binhong69@hotmail.com, hongbin@imb.pumc.edu.cn (Bin Hong).

**Supplemental data**

**Supplemental Figure 1.**


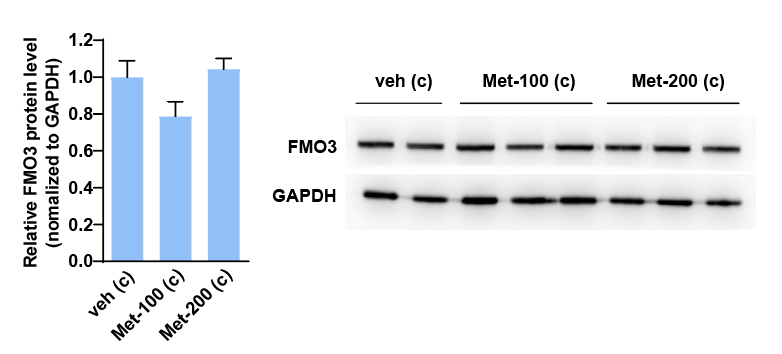


**Supplemental Figure 1.** Effects of metformin on hepatic FMO3 protein level in C57BL/6J mice fed 1% choline diet. FMO3 expression were detected by western blotting assay. Values are presented as means ± SEM. veh (c), n=5; Met-100/200 (c), n =8.

**Supplemental Figure 2.**


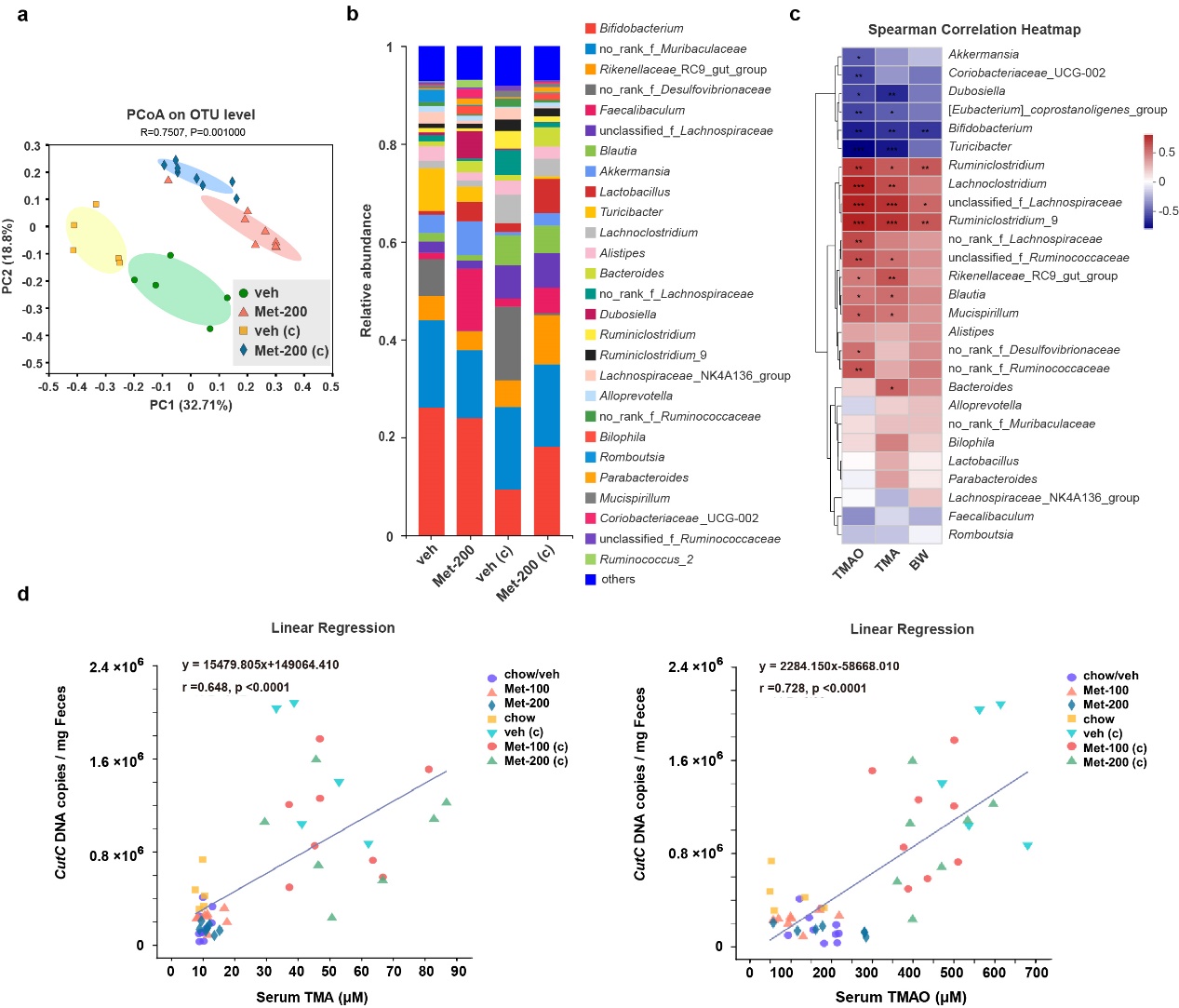


**Supplemental Figure 2.** Effect of metformin and diet on gut microbiota composition of C57BL/6J mice. **a.** Principal coordinate analysis (PCoA) of gut bacterial communities subjected to different treatment. Distances were calculated with bray curtis algorithm. **b.** The composition of dominating genus bar plot. veh, n=5; veh (c), n=5; Met-200, n=8; Met-200 (c), n=8. Others, the relative abundance is less than 1%; f, family; no_rank, the sequence with no clear taxonomic information at the genus level in the database; unclassified, the sequence could not be classified to the genus level in the database. **c.** Heatmap of the Spearman correlation between key phylotypes of gut microbiota and phenotypes (body weight (BW), serum TMA and TMAO levels). Top 27 dominant genera are shown. Red represents a positive association, blue represents a negative association, and white indicates no association (**p*<0.05, ***p*<0.01, ****p*<0.001). **d.** Linear regression analysis of the correlation between the copy number of *cutC* gene and serum TMA and TMAO levels.

**Supplemental Figure 3.**


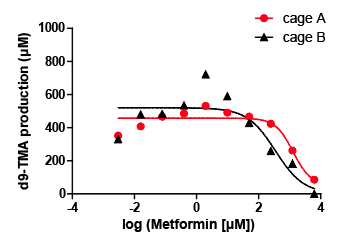


**Supplemental Figure 3**. Metformin treatment inhibited TMA production from choline in gut microbiota *ex vivo*. The microbiota of the feces from chow diet fed C57BL/6J mice (n=5 for each cage, the feces of the same cage were pooled) were used for an *ex vivo* assay. The production of d9-TMA from d9-choline was measured after metformin treatment for 14 h under anaerobic condition.

**Supplementary Methods**

**Animal and treatments**

All animal experiments were performed following the recommendations in the Guide for the Care and Use of Laboratory Animals and were approved by the Institutional Authority for Laboratory Animal Care of Institute of Medicinal Biotechnology. For all experiment, 6- to 8-week-old female C57BL/6J mice (C57BL/6JNifdc, Beijing Vital River Laboratory Animal Technology Co., Ltd) were fed a chow diet or high choline diet. Animals were maintained at a controlled temperature (22 ± 2 °C), with a 12-h light/dark period. The mice were housed individually in cages and had ad libitum access to water. Pentobarbital sodium anesthesia (50 mg/kg of body weight) was administered prior to surgical procedures to maximally minimize suffering. At the end of the experiments, mice were killed by cervical dislocation.

Five to eight mice were applied in each group in accordance with 3R principle (Reduction, Replacement, Refinement involved in the ethical approach applied to animal studies internationally). No statistical methods were used to predetermine sample size. *In vivo* mice studies suggested a group size of at least 5. Mice in all studies were randomized to their particular groups at time of allocation to experimental groups. Investigators performing quantitative analyses of mice serum assays were blinded to group allocation with samples labeled by code only. Investigators were not blinded to mouse group allocation during the performance of animal husbandry requirements for experiments.

**Reagents and antibodies**

Standards for metformin, choline, TMAO, TMA, d9-choline, and d9-TMA were purchased from Sigma-Aldrich (St. Louis, MO, USA). Antibodies against FMO3 was purchased from Abcam (Cambridge, UK, ab126711). Antibodies against GAPDH (TA-08), and horse radish peroxidase (HRP)-conjugated secondary antibodies (ZB-2301, ZB-2305) were purchased from ZSJQ-Bio (Beijing, China)

**HPLC-MS/MS detection of serum TMA/TMAO level**

To eliminate proteins, each 25 μL of serum sample was mixed with 75 μL of 80% acetonitrile. After 30 min of precipitation, supernatant was recovered following centrifugation (14,000 × g, 4 °C, 30 min) and filtrated. To quantify the concentration of TMAO and TMA in each analyte, various concentrations of standards TMAO and TMA were added to control serum to generate calibration curves. The method was validated by calculating the slope variance generated from spiking standards to at least 3 different control serum samples. For TMA analyzation, the TMA standards were ranging between 12.5 µM and 200 µM, and the slope for the standard curve is calculated as 0.042±0.002 (R^2^>0.98 for each curve, CV<10%). The slope for TMAO standard curve is 0.004±0.00038 (R^2^>0.97 for each curve, CV<10%) when the concentrations ranging between 25 µM and 800 µM. Samples were analyzed with LC-MS/MS using an Agilent 6400 Series Triple Quadrupole mass spectrometer (Agilent Technologies, Wilmington, DE, USA) equipped with an electrospray ionization source. Chromatographic separation was performed at a flow rate of 0.25 ml/min on an XBridge™ HILIC column (150 × 2.1 mm, internal diameter of 3.5 μm; WATERS) protected by a flex capillary XBridge™ HILIC guard column (10 × 2.1 mm, internal diameter of 3.5 μm; WATERS). An isocratic elution was generated by mixing acetonitrile (phase A) and water with 10 mM ammonium formate (phase B) at a ratio of 50:50. The capillary voltage was set at +4000 V and heated to 350 °C. Analytes were monitored in positive-ion mode with multiple reaction monitoring (MRM) of precursor and characteristic product-ion transitions of TMAO at m/z 76→58, and TMA at m/z 60→44.

**Quantitation of d9-TMA production from d9-choline**

The d9-TMA producing activity of mice fecal sample was quantified *ex vivo* in anaerobic condition (10% CO_2_ -10% H_2_ -80% N_2_) using AW400SG anaerobic workstation (Electrotek, West Yorkshire, UK). 50 mg of fresh sample was vortexed to suspended in 500 μL of sterile Brucella broth and briefly centrifuged (200× g, 10 s, 4 ºC) to remove solid residues. Then 100 μL of turbid supernatant was inoculated into a capped 96-deep-well plate containing 1 ml of Brucella broth (containing 5% horse blood) supplemented with 1 mM (trimethyl-d9)-choline. The plate was subsequently sealed with sterile foil and incubated anaerobically for 14 h at 37 °C.

Similarly, the d9-TMA producing activity of TMA producing bacteria was determined *in vitro* in anaerobic condition. *Clostridium asparagiforme* DSM 15981, *Clostridium sporogenes* ATCC 19404 and *Escherichia fergusonii* ATCC 35469 were inoculated in the 96-deep-well plate containing 1 ml of same media at an initial concentration of 1×10^6^ CFU/ml and incubated for 24 h at 37 °C. The production of d9-TMA and OD_600_ was detected.

The culture was centrifugated at 4 °C, and the supernatants were collected and filtered through 0.22 μm filter (Shimadazu, Japan). Then the samples were subjected to HPLC-MS/MS detection as mentioned above with precursor and characteristic product-ion transitions of d9-TMA at m/z 69→51. Various concentrations of standards d9-TMA were added to blank broth to generate calibration curve and concentration of d9-TMA in each sample were determined by comparing the peak integrations to that of a calibration curve of d9-TMA.

**Western blot analysis for hepatic FMO3**

Liver samples were homogenized in T-PER tissue protein extraction buffer (Thermo Scientific, Wilmington, DE, USA) by the Fast-Prep Instrument (MP Biochemicals, USA). Protein samples were separated by 10% SDS-PAGE followed by transfer onto a 0.45 PVDF membrane (Millipore, Bedford, MA, USA). The membranes were incubated with primary antibodies against FMO3 (Abcam, USA) or GAPDH (ZSJQ-Bio, Beijing, China), followed by horse radish peroxidase (HRP)-conjugated secondary antibodies (ZSJQ-Bio). The signals were visualized using an enhanced chemiluminescence detection system (Millipore).

**Metagenomic DNA extraction and microbial diversity analysis**

Mice fecal content samples (n=52) were collected and snap frozen at -80 ºC. FastDNA® SPIN kit for Feces and the FastPrep® Instrument (MP Biomedicals, Santa Ana, CA, USA) were used to extract and purify the metagenomic DNA. The concentration of DNA samples was measured by Nanodrop 8000 (Thermo Scientific), the quality and integrity were assessed by gel electrophoresis. Extracted DNA was used as template to amplify the V3-V4 region of the bacterial 16S rRNA gene with barcode-indexed primers 338F (5’-ACTCCTACGGGAGGCAGCAG-3’) and 806R (5’-GGACTACHVGGGTWTCTAAT-3’) by PCR, which was carried out with initial denaturation at 95 °C for 3 min, followed by 27 cycles of denaturation at 95 °C for 30 s, annealing at 55 °C for 30 s, and extension at 72 °C for 45 s, and a ﬁnal extension at 72 °C for 10 min. The quality of PCR products was assessed by electrophoresis on 2% agarose gel and further purified using the AxyPrep DNA Gel Extraction Kit (Axygen Biosciences, Union City, CA, USA). The amplicon libraries were used for diversity and structural comparisons of the bacterial species by Illumina MiSeq platform according to standard protocols (performed by Majorbio Bio-Pharm Technology, Shanghai, China). A total of 2,769,948 valid sequences were obtained. After removal of the barcodes and primers, the qualified reads were clustered into the operational taxonomic units (OTUs) at 97% similarity threshold and was aligned with SILVA132/16S bacteria database for taxonomy information. Bioinformatics analysis were conducted on the Majorbio I-Sanger Cloud Platform (www.i-sanger.com).

**Detection of *cutC* abundance via quantitative real-time PCR (qPCR)**

The *cutC* gene abundances were measured by qPCR using degenerate primers (*cutC*_qF: 5’-TTYGCIGGITAYCARCCNTT-3’, *cutC*_qR: 5’-TGNGGYTCIACRCAICCCAT-3’). Amplification was performed with 25 ng of metagenomic DNA from C57BL/6J mice feces using TransStart Tip Green qPCR SuperMix (TransGen Biotech, China) according to the manufacturer’s instructions. Final primer concentration was 400 nM. An initial 95 ºC for 10 min was followed by 40 cycles of denaturation at 95 ºC for 30 s, annealing at 57 ºC for 20 s, and an extension step at 72 ºC for 20 s. Reactions were performed on the Bio-Rad CFX96 real-time system (Bio-Rad, CA, USA). To generate standard curve, the *cutC* reference gene was amplified using the above primers and two microliter of template DNA, which was a mixture of equal amount of each metagenomic DNA sample from C57BL/6J mice feces. Obtained PCR products were purified (EasyPure® PCR Purification Kit, TransGen Biotech, China) and were quantified by Nanodrop 8000 (Thermo Scientific). Standard curves were generated from dilution of 10^0^ to 10^-6^ ng/µl *cutC* reference gene. The *cutC* gene level of the feces sample was calculated by the standard curve method relative to the weight of each feces sample.
